# Nonlinear Dynamic Mechanical and Impact Performance Assessment of Epoxy and Microcrystalline Cellulose-Reinforced Epoxy

**DOI:** 10.3390/polym16233284

**Published:** 2024-11-25

**Authors:** Mertol Tüfekci

**Affiliations:** 1 Centre for Engineering Research, University of Hertfordshire, Hatfield AL10 9AB, UK; m.tufekci@herts.ac.uk; 2 School of Physics, Engineering and Computer Science, University of Hertfordshire, Hatfield AL10 9AB, UK

**Keywords:** epoxy resin, microcrystalline cellulose (MCC), flexural properties, Charpy impact performance, dynamic mechanical analysis (DMA)

## Abstract

This study focusses on imrpoving the mechanical performance of epoxy resin by reinforcing it with microcrystalline cellulose (MCC). Epoxy composites with varying MCC mass fractions (0.5%, 1%, 1.5%, and 2%) are prepared and characterised to assess the influence of MCC on strain-rate-dependent flexural properties, impact resistance, and nonlinear viscoelastic behaviour. Three-point bending tests at different strain rates reveal that MCC notably increases the flexural strength and leads to nonlinear mechanical behaviour. It is shown that stiffness, strength and elongation at break increase with rising MCC content. Charpy impact tests show improved energy absorption and toughness, while Dynamic Mechanical Analysis (DMA) demonstrates that the materials prepared exhibit increased storage modulus and improved damping across a frequency range. These results indicate that MCC serves as an effective bio-based reinforcement, significantly boosting the strength and toughness of epoxy composites. The findings contribute to the development of sustainable, high-performance materials for advanced engineering applications.

## 1. Introduction

The development of high-performance composite materials represents a critical frontier in materials science, driven by the growing demand for sustainable engineering solutions that permit superior mechanical properties [1,2]. A significant breakthrough in this field has been the incorporation of nanomaterials into polymer matrices, which enables one to have precise control over material behaviour. In this regard, nanocellulose has emerged as a particularly promising candidate among these nanomaterials, distinguished not only by its biodegradability and high aspect ratio, but also by its remarkable mechanical properties and potential for large-scale industrial applications [3,4]. The successful extraction of nanocellulose from several plant sources including hemp [5,6], tea waste [7], rice husk [8], and agricultural residues [9] demonstrates its potential to change sustainable composite manufacturing while addressing global challenges in waste management and resource utilisation.

Nanocellulose-reinforced composites are some good alternatives to traditional materials, with applications in structural, aerospace and biomedical fields [10,11,12,13,14,15]. Integration of nanocellulose in polymer matrices like epoxy has significantly increased tensile strength, modulus, toughness, and damping properties [10,16,17]. Other recent applications include membrane technology for water treatment and development of biocomposites with superior thermal and mechanical characteristics [18,19,20,21,22,23]. In environmental applications, nanocellulose-based aerogels have also attracted attention due to their high porosity and surface area [24,25,26].

Microcrystalline cellulose (MCC), a versatile and widely studied nanocellulose form, has shown promise as a reinforcement in epoxy-based composites [27,28,29,30]. Research has expanded MCC’s applicability by extracting it from waste materials, enhancing sustainability, and employing surface modification with silane compounds to improve its compatibility with epoxy resins, as well as its thermal stability and mechanical performance [28,31,32,33]. Incorporating tailored MCC with improved crystallinity and purity further enhances these properties [7,34]. Other novel methods such as paraffin wax encapsulation have also improved dispersion, and MCC has been combined with additives to boost mechanical strength and flame-retardant properties [27,30].

Nanocellulose-based materials like cellulose nanofibers, cellulose nanocrystals, and bacterial cellulose are some alternatives that can be beneficial for improving the mechanical and thermal properties of polymer composites [4,10,11,18,19,22,23,35,36]. These materials can form entangled networks within the polymer matrix, increasing reinforcement [37,38,39]. Studies show that adding CNFs to epoxy resin improves modulus, strength, and toughness [10,16,17,20,40,41].

Dynamic mechanical analysis (DMA) is a critical method for characterising viscoelastic properties in nanocellulose-reinforced composites [42,43,44,45]. These composites exhibit increased storage modulus and damping factor, indicating enhanced stiffness and energy dissipation [10,39,46,47,48]. MCC’s surface properties, geometry, and aspect ratio influence mechanical outcomes, especially through mechanisms like fibre-matrix adhesion [22,38,49,50]. Studies have also explored nonlinear mechanical properties in epoxy-MCC composites, finding improved stiffness and damping behaviour [16]. Modelling approaches such as the Generalised Maxwell Model and mean-field homogenisation are shown to be accurate to predict the effects of the reinforcements on the mechanical behaviour of materials [43,44,45,51].

MCC-reinforced composites show potential in various applications, from structural components to biocompatible medical devices. In water treatment, MCC-based membranes exhibit improved permeability and selectivity [18,19,21,36,52,53,54]. MCC’s biocompatibility and biodegradability as well as sustainability also make it ideal for wound dressings, tissue scaffolds, and drug delivery [11,22,55,56]. Structural applications include additively manufactured parts, where nanocellulose improves mechanical properties [46,47,57,58].

Strain-rate-dependent properties, crucial for performance under dynamic loading, are also improved by nanocellulose, making these composites useful for high-energy absorption applications [59,60,61]. Research on the deformation of composites, such as ultra-high-molecular-weight polyethylene fibre-reinforced composites and snap-cure epoxy resins, provides essential data on crashworthiness [59,60]. Progress in sustainable fabrication, including cellulose extraction from waste, contribute to reducing environmental impact [7,26,32,35,37]. Challenges include scaling production while maintaining quality and consistency, standardising characterisation techniques, and understanding the relationship between nanoscale and macroscale performance [35,37].

While many studies review bio-fibre composites [1,2,38,62], specific investigation into MCC-reinforced epoxy remains limited. To address gaps in existing research, this study examines the reinforcement of epoxy resin with MCC to improve its mechanical properties. Epoxy composites are prepared with various MCC mass fractions $m_{f}$ (0.5%, 1%, 1.5%, and 2%) using a standardised manufacturing process to ensure uniform dispersion and improve the chances of strong interfacial bonding between the MCC and the epoxy matrix. A comprehensive mechanical characterisation is carried out throug DMA, three-point bending and Charpy impact tests. DMA is used to characterise the viscoelastic properties of the epoxy composites over a range of frequencies, providing a detailed understanding of the stiffness and damping behaviour resulting from MCC incorporation. Also, three-point bending tests are performed at different strain rates, aimed at investigating the strain-rate-dependent flexural properties and assessing the nonlinear mechanical behaviour introduced by the MCC reinforcement. Charpy impact tests are also performed to evaluate the impact resistance and energy absorption capacity. The novelty of this study lies in the systematic and thorough evaluation of MCC as a reinforcement in epoxy composites, particularly its effects on strain-rate sensitivity and dynamic mechanical properties. While previous studies have explored the use of nanocellulose in epoxy resins, limited data exist on the specific influence of MCC on the nonlinear mechanical behaviour and viscoelastic properties of epoxy matrices. This research addresses that gap, providing more understanding of the effects of the MCC reinforcement and demonstrating its potential to significantly enhance the performance of epoxy composites. These findings contribute to the development of sustainable, high-performance composite materials and provide information for tailoring bio-based polymer composites in various engineering applications.

## 2. Material Sample Preparation

The epoxy resin MGS^®^ L 285 and hardener MGS^®^ H 285 by Hexion are used as the matrix materials, consistent with previously published study [16,40]. MCC supplied by Sigma-Aldrich (St. Louis, MO, USA) (Product no: 310697) with an average particle size of 20 μm is used as the reinforcement. The MCC is added to the resin at mass fractions ($m_{f}$) of 0.5%, 1%, 1.5%, and 2%, similar to studies in the literature [16,40].

Weighing is done using a Mettler-Toledo Standard Level Balance ME Precision Balance with a capacity of 5200 g and precision between 0.001–0.01 g. In each material preparation, 500 g of material is produced.

### 2.1. Preparation of Epoxy

The epoxy resin and hardener are mixed until colour homogeneity is achieved, as described previously [40]. Mixing is done slowly to avoid introducing air bubbles. The mixture is poured into moulds and placed in a vacuum chamber for 2 h. Afterward, the moulds are left under ambient conditions for 24 h to cure, followed by post-curing in an incubator at 60 °C for 15 h.

### 2.2. Preparation of MCC-Reinforced Epoxy

The MCC particles are dried in an oven at 60 °C for at least 8 h to minimise moisture content. They are then blended into the resin component of the epoxy using an electromagnetic mixer at 50 °C and 100 rpm for at least 2 h. An ultrasonic homogeniser is employed to ensure uniform particle distribution and prevent agglomeration. Temperature is monitored to prevent any adverse chemical reactions due to excessive heating.

After mixing, the mixture is degassed in a vacuum chamber for at least one hour. The hardener is then slowly mixed in until colour homogeneity is achieved, minimising air bubble formation. The final mixture is poured into moulds and placed in a vacuum chamber for two hours. The same curing and post-curing processes are followed as for the pure epoxy samples.

The mass composition of the composites is presented in Table 1.

## 3. Experimental Characterisation

To evaluate the mechanical properties of the epoxy and MCC-reinforced epoxy composites, a series of tests are conducted. These tests include three-point bending tests, Charpy impact tests, and DMA. The purpose of these experiments is to understand the strain-rate dependence, impact resistance, and viscoelastic behaviour of the materials. The following sections detail the methodologies employed for each test.

### 3.1. Dynamic Mechanical Analysis

**Figure 1:**
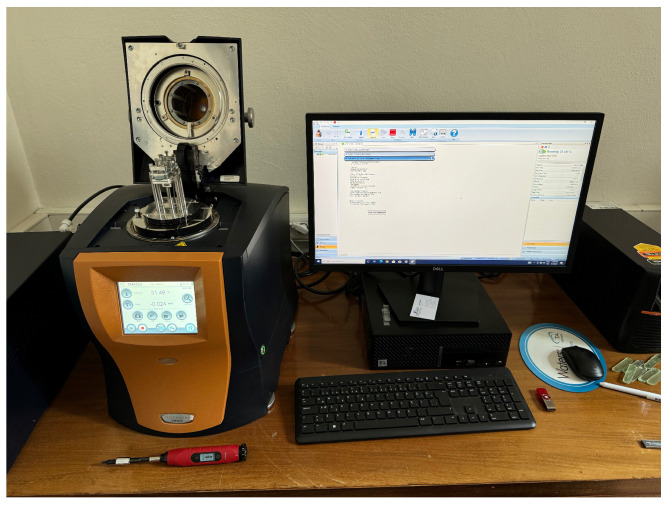
DMA test setup with dual cantilever fixture and specimen dimensions.

DMA is utilised to characterise the viscoelastic properties of the composites, providing information on stiffness, damping behaviour, and how these properties change with frequency and strain amplitude. The tests are performed using a TA Instruments (New Castle, DE, USA) DMA 850 with the dual cantilever fixture, as illustrated in Figure 1. Specimens measure 70 mm in length, 14 mm in width, and 3 mm in thickness.

During the tests, the samples are subjected to harmonic oscillations with frequencies up to 150 Hz and strain amplitudes reaching 0.1%. The dual cantilever configuration is chosen to ensure adequate stiffness and to minimise compliance effects. Clamps are tightened to a torque of 0.9 N·m (8 in-lbs) to prevent slippage and ensure accurate measurement of material damping without significant contributions from friction at the interfaces [63].

All tests are conducted at room temperature to maintain consistent thermal conditions, as the mechanical properties of polymers are sensitive to temperature variations [64]. Prior to testing, specimens are allowed to equilibrate in the DMA chamber for five minutes to eliminate any thermal gradients and to stabilise the material after any handling or preparation steps. Each test is repeated at least three times to verify the repeatability and reliability of the results.

### 3.2. Three-Point Bending Tests

**Figure 2:**
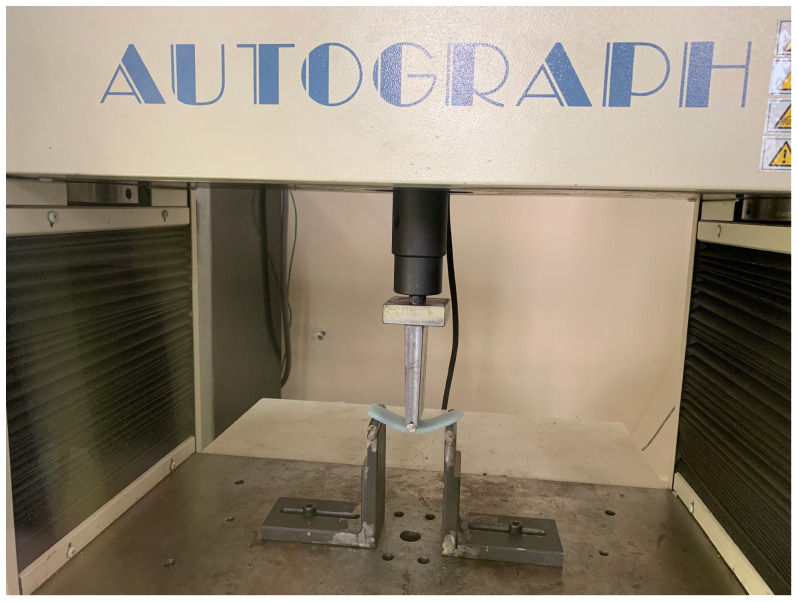
Three-point bending test setup and specimen dimensions.

The three-point bending tests are performed to investigate the strain-rate-dependent mechanical behaviour of the composites, which are expected to exhibit nonlinear characteristics due to the polymeric nature of the epoxy matrix. The tests are carried out in accordance with the ASTM D7264 standard [65] using a Shimadzu (Kyoto, Japan) AG-IS 50 kN universal testing machine, as shown in Figure 2. Samples are subjected to three different strain rates: $0.01\min^{-1}$, $0.05\min^{-1}$, and $0.1\min^{-1}$, covering a range from quasi-static to higher deformation speeds. This range is selected based on equipment capabilities and to align with previous studies for comparative purposes [40,66,67].

The specimens are prepared according to the dimensional requirements specified in the relevant standard. To ensure consistency and reliability of the results, five specimens are tested for each material group across all tests. The effects of MCC reinforcement on the flexural properties are assessed by comparing the results with those of the unreinforced epoxy samples.

### 3.3. Charpy Impact Tests

**Figure 3:**
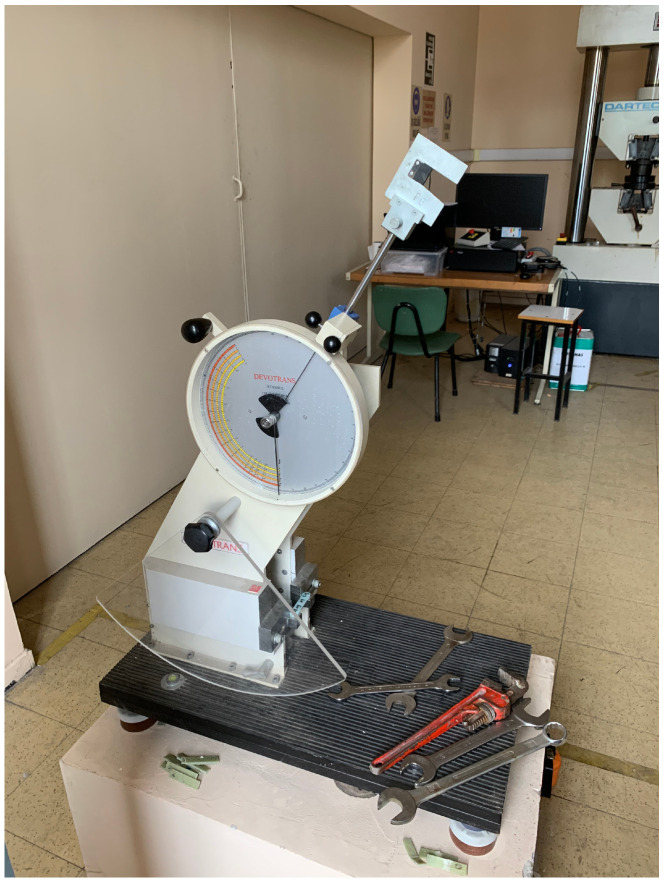
Charpy impact test setup.

To evaluate the impact resistance and fracture energy of the composites, Charpy impact tests are conducted according to ASTM D6110 [68]. The testing apparatus is shown in Figure 3. Five specimens from each material group, including both the pure epoxy and MCC-reinforced composites, are tested to obtain statistically significant results. The impact tests aim to determine how the addition of MCC affects the ductility and energy absorption capabilities of the epoxy matrix.

## 4. Results and Discussion

### 4.1. Dynamic Mechanical Analysis

**Figure 4:**
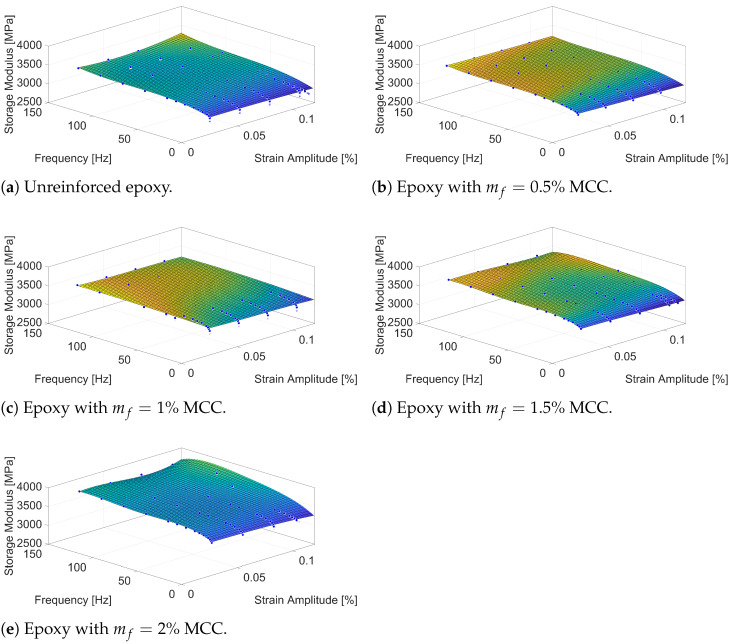
Storage modulus of epoxy samples with various MCC mass fractions ($m_{f}$).

The analysis of the storage modulus ($E^{\prime }$) results for epoxy samples reinforced with MCC at various mass fractions ($m_{f}$), shown in Figure 4, reveals the correlation between reinforcement concentration and the dynamic mechanical behaviour of the composite. It is important to highlight that MCC, with a modulus of elasticity significantly higher than that of unreinforced epoxy, serves as a stiffening phase within the polymer matrix. However, the effectiveness of this reinforcement is influenced by stress interactions at the interface and the mass fraction of MCC in the composite, particularly as higher concentrations create more significant values stress.

For the unreinforced epoxy sample (Figure 4a), the storage modulus remains relatively flat across the entire frequency range, maintaining a value around 3500 MPa. This consistency indicates that unreinforced epoxy exhibits minimal strain- and/or frequency-dependent stiffness changes, likely due to the homogeneous nature of the polymer matrix, which does not have any particulate inclusions to alter its stiffness under various conditions. Unreinforced epoxy behaves primarily as a quasi-linear viscoelastic material, whose stiffness is stable across the frequency range.

Upon the inclusion of MCC at a mass fraction of $m_{f}=0.5\%$ (Figure 4b), the storage modulus values increase slightly across the frequency spectrum compared to unreinforced epoxy. This behaviour reflects the introduction of a stiffer reinforcement phase into the polymer matrix, although at this concentration, the distribution of MCC particles may not yet form a highly synergistic load-bearing network. At low strain amplitudes, the modulus shows a modest frequency-dependent increase, suggesting that the MCC particles contribute to stiffness enhancement even at this early stage.

As the mass fraction of MCC increases to $m_{f}=1\%$ (Figure 4c), the storage modulus begins to show a noticeable variation with increasing strain amplitude. At this concentration, stiffness increases are evident as the MCC particles start to carry a bigger portion of the load, which enhances the overall load transfer from the matrix to the reinforcements. The interactions between the matrix and MCC particles contribute to the formation of effective stress transfer sites, improving the composite’s ability to resist deformation under applied loads. Additionally, the frequency dependence becomes more pronounced at this concentration, with higher storage modulus values observed at elevated frequencies, indicating frequency-related stiffening behaviour. However, at higher strain amplitudes, a slight reduction in stiffness is observed, possibly due to localised stress concentration effects or partial debonding at the particle-matrix interface.

When the MCC content rises to $m_{f}=1.5\%$ (Figure 4d), the effects of reinforcement become more obvious. The stiffness increases more substantially due to the higher density of MCC particles within the matrix, which facilitates greater interaction among the particles and the matrix. While the stiffness remains stable at low strain amplitudes and frequencies, the frequency-dependent increase in stiffness becomes more evident, reflecting improved energy storage efficiency at higher frequencies. Softening behaviour becomes more noticeable at higher strain amplitudes, which may stem from increased stress concentrations as neighbouring MCC particles interact, leading to elevated combined stresses within the matrix. However, the overall trend remains upwards, with increased modulus values compared to lower MCC concentrations, particularly at low strain amplitudes.

At higher mass fractions $m_{f}=2\%$ (Figure 4e), the particles are more closely positioned within the epoxy matrix, leading to increased stress concentration, particularly at higher strain amplitudes. This packing effect, combined with interfacial interactions, enhances stiffness overall, particularly at low strain amplitudes and high frequencies, as evident in the consistently elevated modulus values. However, at very high strain amplitudes, the interaction of stress concentration zones may contribute to localised plastic deformation, which can slightly reduce load transfer efficiency in specific regions. Nevertheless, the frequency-dependent stiffening remains strong at this concentration, indicating that the MCC reinforcement continues to enhance energy storage within the composite.

The absence of a very strong frequency-dependent stiffening across all MCC mass fractions suggests that the dynamic mechanical properties of the epoxy composites are primarily controlled by the matrix itself, with MCC providing a modest yet steady reinforcement effect overall. However, the observed increase in storage modulus with frequency for all MCC contents highlights the role of reinforcement in improving/increasing energy storage capabilities under cyclic loading. This is particularly evident in the low strain amplitude region, where the storage modulus remains nearly constant across the frequency spectrum, implying that the introduction of MCC does not significantly alter the viscoelastic nature of the epoxy under cyclic loading but does enhance its stiffness.

It is also worth considering that at higher frequencies, the polymer chains in epoxy may not have enough time to relax, which could enhance the modulus slightly. The inclusion of MCC makes this effect more noticeable by restricting polymer compliance further, resulting in more significant frequency-dependent stiffening compared to the unreinforced matrix. The lack of notable frequency-dependent stiffening at lower MCC concentrations supports the hypothesis that while MCC increases overall stiffness, its effects become more pronounced at higher contents and frequencies. This trend underscores the importance of both frequency and strain amplitude in evaluating the dynamic performance of MCC-reinforced epoxy composites.

**Figure 5:**
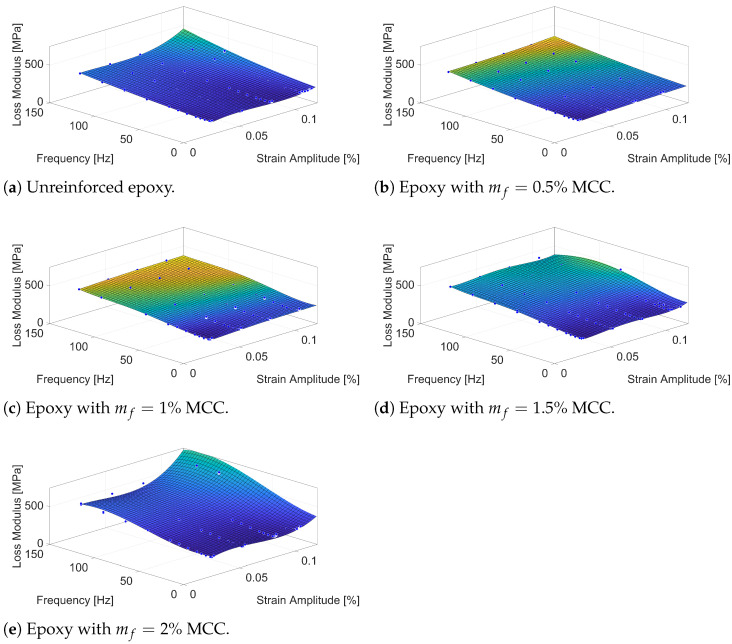
Loss modulus of epoxy samples with various MCC mass fractions ($m_{f}$).

The results presented in Figure 5 illustrate the variation in loss modulus for epoxy composites reinforced with MCC at mass fractions ($m_{f}$) of 0.5%, 1%, 1.5%, and 2%, compared to the unreinforced epoxy. The loss modulus ($E^{\prime \prime }$) indicates the material’s ability to dissipate energy as heat during cyclic loading, which is crucial for understanding the viscoelastic behaviour and damping properties of the composites.

The unreinforced epoxy (Figure 5a) displays a relatively uniform loss modulus across the frequency and strain amplitude spectrum, showing minimal energy dissipation and low damping properties, as expected for a typical thermosetting polymer without reinforcement. The low values of $E^{\prime \prime }$ suggest that the free movement of polymer chains results in minimal internal friction, leading to reduced energy dissipation. Again, the unreinforced epoxy displays quasi-linear material properties.

When MCC is introduced at 0.5% ($m_{f}=0.5\%$, Figure 5b), the loss modulus begins to increase, particularly at higher frequencies and strain amplitudes. The slight rise in $E^{\prime \prime }$ suggests enhanced energy dissipation due to the presence of interfaces between the epoxy matrix and the MCC particles. These interfaces generate interfacial friction, increasing internal resistance against polymer chain mobility during deformation, thereby enhancing damping. At this low level of reinforcement, polymer chain mobility is still relatively high, but the introduction of MCC creates localised regions of interaction, increasing internal friction and energy dissipation. The presence of particles also change the strain field in the epoxy matrix which also contributes to the change of material damping capacity measured.

As the MCC content rises to 1% ($m_{f}=1\%$, Figure 5c), there is a more noticeable increase in the loss modulus, especially at elevated frequencies. This reflects a greater contribution of MCC particles to the energy dissipation mechanisms. The increased loss modulus $E^{\prime \prime }$ suggests that MCC particles at this concentration begin to act more efficiently as sites of internal friction, leading to a more effective restriction of polymer chain movement. The polymer chains interact more intensively with the rigid MCC particles, which increases energy dissipation due to increased internal friction and localised stress concentrations. At this reinforcement level, the distribution of MCC particles appears sufficient to enhance interfacial friction and restrict polymer mobility without introducing significant limitations due to particle interactions.

With 1.5% MCC ($m_{f}=1.5\%$, Figure 5d), the loss modulus exhibits a significant increase across all frequencies, indicating that the reinforcement content now plays a more dominant role in restricting molecular mobility and enhancing viscoelastic properties. The epoxy matrix becomes more constrained, and MCC particles establish a greater number of internal friction interfaces, where energy dissipation becomes increasingly effective. Also, the strain field is more significantly changed. Therefore, the material displays a different characteristic for vibration damping. At higher strain amplitudes, interactions between MCC particles and the epoxy matrix lead to increased internal friction and energy dissipation, contributing to the overall damping capacity.

At 2% MCC ($m_{f}=2\%$, Figure 5e), the loss modulus reaches its peak, demonstrating the most significant energy dissipation capabilities. The higher loss modulus $E^{\prime \prime }$ values at lower and medium frequencies suggest that the MCC particles form a more interconnected network within the epoxy matrix, further restricting polymer chain movement. However, at very high frequencies, the increase in the loss modulus begins to taper off. This saturation effect likely arises due to the increased interaction between MCC particles at higher concentrations, where neighbouring stress fields begin to overlap. These interactions can create localised zones of stress concentration, changing the strain field and strain energy, which may alter the efficiency of energy dissipation. At very high frequencies, the polymer chains in these regions may experience restricted movement due to overlapping stress fields, potentially limiting further gains in energy dissipation. Instead of uniformly dissipating energy, the system may favour energy storage in these regions, contributing to the observed levelling off of the loss modulus.

The observed behaviour in the loss modulus across the various MCC content levels can be explained by several key physical mechanisms. Interfacial friction between the epoxy matrix and MCC particles plays a major role, where polymer chains experience restricted movement around rigid inclusions, leading to increased heat generation and energy dissipation. As MCC content increases, stress concentration zones are formed around the particles, which generate micro-deformations that contribute to energy dissipation. These zones facilitate the conversion of mechanical energy into heat, raising the loss modulus values. However, at higher reinforcement levels, the increased interaction between neighbouring stress concentration zones can limit the overall dissipation effectiveness, particularly at elevated frequencies as they change the strain field and energy.

**Figure 6:**
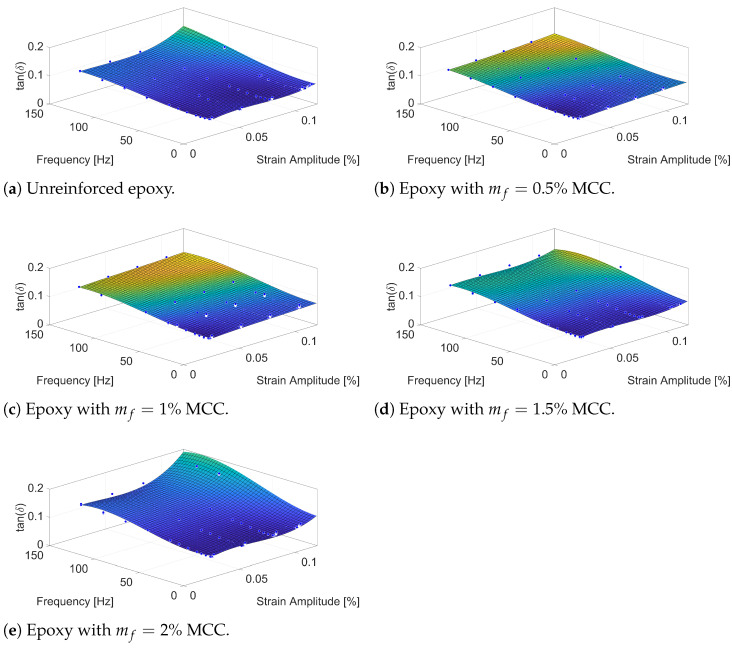
tan(δ) of epoxy samples with various MCC mass fractions ($m_{f}$).

The results in Figure 6 illustrate the variation in damping factor tan(δ) for epoxy composites reinforced with MCC at various mass fractions ($m_{f}$) of 0.5%, 1%, 1.5%, and 2%, across a range of frequencies and strain amplitudes. As the damping factor tan(δ) is the ratio of the loss modulus $E^{\prime \prime }$ to the storage modulus $E^{\prime }$, its trends reflect the balance between energy dissipation and energy storage within the material under cyclic loading.

For the unreinforced epoxy sample (Figure 6a), damping factor tan(δ) remains nearly constant across both frequency and strain amplitude ranges, with values around 0.1. This behaviour indicates minimal energy dissipation and highlights the stable viscoelastic response of the pure epoxy matrix, which lacks significant internal friction or reinforcing interfaces.

At 0.5% MCC (Figure 6b), a slight increase in damping factor tan(δ) is observed, particularly at higher frequencies and strain amplitudes. This reflects an improvement in energy dissipation due to the introduction of MCC, which creates internal interfaces where frictional and strain-energy-driven mechanisms contribute to the loss modulus. However, with MCC content still low, the storage modulus $E^{\prime }$ dominates, limiting the overall increase in damping factor tan(δ).

When the MCC content rises to 1% (Figure 6c), damping factor tan(δ) increases more noticeably across the entire spectrum, particularly at higher frequencies. This trend suggests that interfacial friction and localised matrix deformation become more effective at dissipating energy as the MCC content increases. The increased damping behaviour reflects a higher contribution of loss modulus $E^{\prime \prime }$ due to increased interactions between MCC particles and the surrounding matrix.

With 1.5% MCC (Figure 6d), damping factor tan(δ) continues to rise across both frequency and strain amplitude ranges, indicating further improvements in energy dissipation. The higher MCC content enhances the interactions between particles and the matrix, contributing to a significant increase in loss modulus $E^{\prime \prime }$. At this concentration, damping becomes more pronounced at higher strain amplitudes, where polymer deformation around the stiffer inclusions is greater, further increasing energy dissipation.

At 2% MCC (Figure 6e), damping factor tan(δ) exhibits a reduced rate of increase, particularly at higher frequencies. This levelling off of tan(δ) can be attributed to a more substantial increase in storage modulus $E^{\prime }$ compared to loss modulus $E^{\prime \prime }$, reflecting a shift towards greater stiffness relative to energy dissipation. While the higher MCC content continues to restrict polymer chain mobility and enhance energy dissipation, the material increasingly behaves as a stiffer composite, where energy storage becomes more dominant.

Overall, the trends in damping factor tan(δ) demonstrate a complex interplay between the loss modulus loss modulus $E^{\prime \prime }$ and storage modulus $E^{\prime }$. The addition of MCC improves the composite’s damping behaviour, particularly at moderate reinforcement levels, where loss modulus $E^{\prime \prime }$ increases significantly. At higher MCC contents, the increase in storage modulus $E^{\prime }$ begins to outpace that of loss modulus $E^{\prime \prime }$, leading to a more elastic response and reduced frequency sensitivity of the damping factor tan(δ) at the highest concentrations.

MCC reinforcement improves the damping properties of epoxy composites, as demonstrated by the trends in damping factor tan(δ) and the interplay of storage modulus $E^{\prime }$ and loss modulus $E^{\prime \prime }$ in this study. The observed behaviour aligns with findings in the literature. For example, ref. [27] reported that wax-encapsulated MCC enhanced dynamic mechanical properties by improving both energy dissipation and storage modulus. Similarly, ref. [69] observed that higher MCC content led to increased storage modulus and reduced thermal expansion, contributing to enhanced damping characteristics. In hybrid MCC/liquid rubber composites, ref. [70] found that effective energy dissipation at the interface significantly improved dynamic mechanical behaviour.

The results in this study demonstrate that as MCC content increases, both storage modulus $E^{\prime }$ and loss modulus $E^{\prime \prime }$ are increased, particularly at lower strain amplitudes and moderate frequencies, leading to improved damping properties. At higher MCC content, the increased interfacial area and interaction between MCC particles contribute to improved energy dissipation, as reflected in the trends of damping factor tan(δ). However, at very high frequencies, the effect of MCC reinforcement on damping factor tan(δ) diminishes, suggesting a shift towards more elastic behaviour due to the altered balance between energy storage and dissipation. This frequency dependence is less pronounced at higher MCC concentrations, consistent with reduced chain mobility and a more constrained matrix structure.

These findings provide further confirmation of the role of MCC in affecting the dynamic mechanical properties of epoxy composites. The improved damping behaviour observed in this study supports previous conclusions in the literature while revealing more on the characteristics of the materials particularly focusing on the strain amplitude and frequency dependencies of MCC-reinforced epoxy composites.

### 4.2. Three-Point Bending Tests

**Figure 7:**
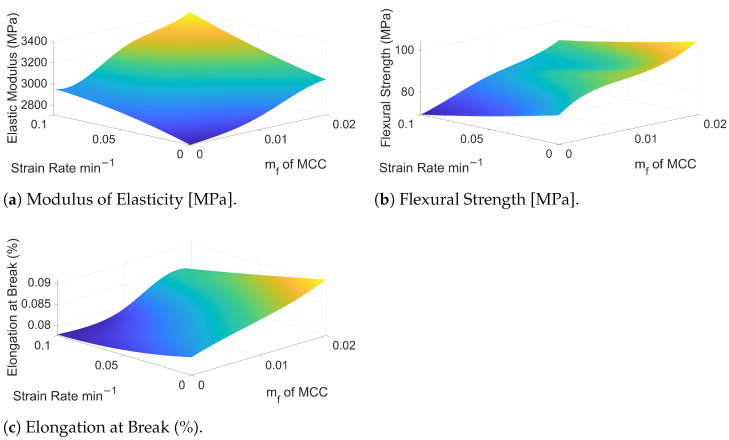
Surface plots showing the dependence of modulus of elasticity, flexural strength, and elongation at break on MCC mass fraction and strain rate.

The results of the analysis are shown in Figure 7, which presents the modulus of elasticity, flexural strength, and elongation at break for various MCC mass fractions and strain rates. The mechanical response of the epoxy-MCC composites exhibits distinct trends depending on both the reinforcement content and the applied strain rate.

The modulus of elasticity of the epoxy composites, shown in Figure 7a, demonstrates a clear upward trend with increasing MCC mass fraction and strain rate. At a static strain rate and without MCC (plain epoxy), the stiffness starts around 2700 MPa. As the mass fraction of MCC increases to 2%, the modulus rises steadily to approximately 3400 MPa at higher strain rates.

This increase in stiffness is primarily related to the inherent high modulus of MCC compared to the epoxy matrix. MCC, being a cellulose-based reinforcement, possesses a crystalline structure and a higher modulus of elasticity compared to epoxy, that provides rigidity, improving the load-bearing capacity of the composite as the stress in the material is transferred to the particles from the epoxy matrix. When MCC is introduced into the epoxy matrix, it restricts the mobility of the polymer chains, thereby increasing the composite’s overall stiffness as the load is increasingly carried by the stiff MCC particles with more MCC present in the matrix.

Also, at higher strain rates, the rapid deformation of the material reduces the time available for the epoxy matrix to go through viscoelastic relaxation. This limited relaxation increases the matrix’s effective stiffness, permitting it to transfer the stress to the MCC reinforcementsmore efficiently. Therefore, the overall mechanical response of the composite becomes stiffer.

Flexural strength, given in Figure 7b, follows a similar trend to that of the modulus of elasticity, showing an increase with both MCC mass fraction and strain rate. Initially, the flexural strength of pure epoxy is approximately 83 MPa, and with the addition of 2% MCC, the flexural strength increases to about 104 MPa, particularly at higher strain rates.

The rise in flexural strength is primarily due to the load-carrying capabilities of the MCC reinforcements and their ability to stop crack propagation. MCC particles act as sites that inhibit crack growth and delay the propagation of cracks through the matrix. MCC particles also change the stress field within the composite, which also impacts the crack initiation and propagation. Additionally, the significant magnitude of the interfacial bonding area between MCC and the epoxy matrix ensures effective load transfer from the matrix to the MCC particles, enabling the reinforcement phase to contribute more to the composite’s overall strength. These mechanisms collectively improve the composite’s ability to withstand higher flexural loads before failure.

Moreover, the increase in flexural strength with strain rate can be related to the behaviour of the epoxy matrix and its interaction with the MCC reinforcements under dynamic loading. At higher strain rates, the material undergoes rapid deformation, which limits the extent of viscoelastic relaxation in the matrix. This reduced relaxation time increases the matrix’s stiffness and enhances its ability to transfer the applied load to the MCC reinforcements more effectively. The stiff MCC particles, in turn, act as barriers to crack propagation, thereby increasing the material’s resistance to failure.

Elongation at break, presented in Figure 7c, exhibits a distinct dependence on both MCC mass fraction and strain rate. For pure epoxy, the elongation at break is approximately 0.082%. With the addition of MCC, the elongation at break decreases slightly at lower strain rates, dropping to around 0.072% for higher MCC contents. This reduction reflects the increased brittleness introduced by the stiff MCC particles, which restrict the matrix’s ability to deform plastically.

At higher strain rates, the elongation at break shows a notable recovery, particularly at higher MCC contents. For example, at a strain rate of $0.1\min^{-1}$, elongation at break increases to approximately 0.091% for composites with 2% MCC. This recovery can be attributed to the reduced time for strain localisation and crack propagation under dynamic loading conditions, which delays the failure of the material. Despite this partial recovery, the overall ductility of MCC-reinforced composites remains lower than that of pure epoxy, as the stiff reinforcements continue to constrain deformation.

While the modulus of elasticity and flexural strength increase consistently with MCC content due to mechanisms such as stress transfer from the matrix to the MCC particles, restricted polymer chain mobility, and the overall increased stiffness provided by the reinforcements, the elongation at break reveals a more complex characteristic. At lower strain rates, the stiff MCC particles reduce polymer chain mobility and localise strain energy, which limits the ability of the material to deform plastically, leading to reduced ductility. Conversely, at higher strain rates, the rapid loading reduces the time available for crack initiation and propagation, allowing the material to sustain greater deformation before failure. These trends reflect the combined effects of reinforcement, strain rate-dependent viscoelastic behaviour, and the interaction between the matrix and the MCC particles under dynamic loading conditions.

### 4.3. Charpy Impact Tests

**Figure 8:**
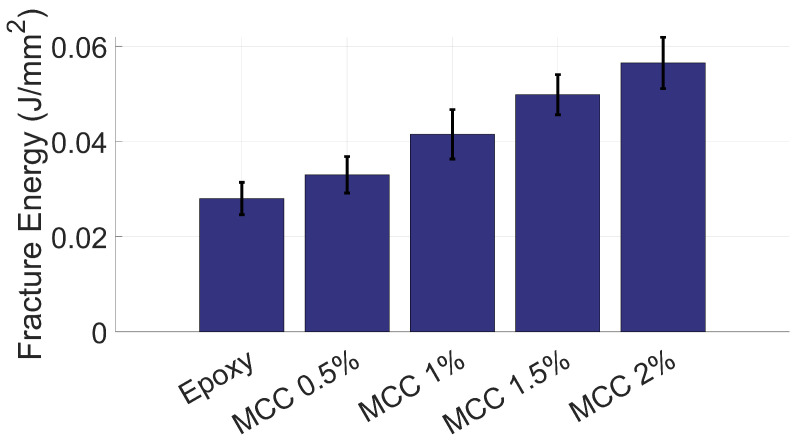
Charpy impact test results.

The bar chart in Figure 8 presents the fracture energy per unit cross-sectional area for the epoxy-based composites reinforced with MCC at different mass fractions. The results demonstrate that fracture energy increases with the addition of MCC, which is consistent across all reinforcement percentages. Unreinforced epoxy, represented by the first bar, exhibits the lowest fracture energy. This can be explained by the inherent brittleness of unreinforced epoxy, which limits its ability to absorb significant amounts of energy before failure.

With the addition of 0.5% MCC, the fracture energy improves noticeably. This rise is related to the effects of MCC reinforcement, which provides resistance to crack propagation, and the effectively transferred load from the epoxy matrix to the MCC particles. As the MCC content increases to 1%, 1.5%, and 2%, a continuous improvement in fracture energy is observed. This is likely due to more effective stress transfer at the matrix-particle interface and the ability of the MCC particles to act as crack stoppers, blunting or deflecting crack fronts and enhancing toughness through interaction among nearby stress concentration points.

From a mechanics standpoint, the inclusion of MCC—which has a significantly higher modulus of elasticity compared to the epoxy matrix—results in an overall stiffer composite. This enhanced stiffness may aid in distributing and absorbing the stress applied during impact, leading to greater energy absorption before fracture. Additionally, mechanisms such as debonding of MCC particles from the epoxy matrix, combined with the interaction of localised stress concentrations, may lead to plastic deformation of the matrix around these particles, further contributing to the increase in fracture energy. As MCC content increases, these mechanisms become more pronounced, allowing more efficient energy dissipation through particle-matrix debonding, plastic deformation, and microcrack deflection.

MCC reinforcement improves the impact resistance of epoxy composites. For example, ref. [71] reported a 28.3% increase in impact energy in MCC-reinforced jute/epoxy composites. Similarly, ref. [29] demonstrated that incorporating hyperbranched aromatic polyamide-treated MCC improved impact strength by 83.4%. This improvement is related to stronger fibre-matrix adhesion, which improves energy absorption and impact toughness. The findings of this study, showing a substantial increase in impact resistance with higher MCC content, align well with these earlier observations that are found in the literature, further reinforcing the role of MCC in improving the impact toughness and overall performance of epoxy-based composites.

## 5. Conclusions

This study presents a detailed examination of the nonlinear dynamic mechanical and impact performance of epoxy composites reinforced with MCC. By evaluating epoxy composites with varying MCC mass fractions (0.5%, 1%, 1.5%, and 2%), this study provides detailed observations on how MCC affects the mechanical properties, contributing to the development of sustainable and high-performance materials.

- **DMA**: The inclusion of MCC significantly affects the viscoelastic properties of the epoxy matrix. An overall increase in storage modulus is observed with MCC content, indicating increased stiffness, particularly at higher mass fractions. The loss modulus and damping factor analysis show that MCC improves energy dissipation, particularly through interfacial friction mechanisms. However, at higher concentrations, localised stress interactions reduce energy dissipation efficiency (damping factor) under specific conditions.
- **Flexural Properties**: The three-point bending tests show that both the modulus of elasticity and flexural strength increase with MCC reinforcement, particularly at higher strain rates. The stiffening effect of MCC and its ability to efficiently transfer stress within the composite contribute to improved load-bearing capacity. While elongation at break decreases with some low level of MCC content, it recovers with higher MCC content. Additionally, at higher strain rates, the elongation at break values increase slightly.
- **Impact Resistance**: The Charpy impact tests show that MCC increases the fracture energy and impact resistance of the epoxy composites, with the highest fracture energy observed in the samples containing 2% MCC. This rise is related to the ability of MCC particles to arrest crack propagation and improve toughness as well as stiffen the composite material.

The research demonstrates that MCC is an effective reinforcement for epoxy composites, capable of improving stiffness, damping, flexural strength, and impact resistance while introducing nonlinear mechanical behaviour. This research highlights the potential of MCC as a sustainable reinforcement in polymer composites, promoting the development of greener, high-performance materials suitable for advanced engineering applications.

Future work should focus on tailoring the spatial arrangement and interaction of MCC particles within the epoxy matrix and investigating surface treatments to further enhance interfacial bonding. Additionally, studying the long-term durability of MCC-reinforced epoxy composites under various environmental conditions will provide greater understanding about their practical application in structural and impact-resistant components.

## Figures and Tables

**Table 1:** Composition by mass of materials depending on the MCC reinforcement (measured in grams).

Mass Fraction ($m_{f}$)	Epoxy Resin (g)	Hardener (g)	MCC (g)
0.005	355.36	142.14	2.50
0.01	353.57	141.43	5.00
0.015	351.79	140.71	7.50
0.02	350.00	140.00	10.00

## Data Availability

No data is generated for this research apart from the data presented in the paper.
